# Effect of Intermittent Microwave Volumetric Heating on Dehydration, Energy Consumption, Antioxidant Substances, and Sensory Qualities of Litchi Fruit during Vacuum Drying

**DOI:** 10.3390/molecules24234291

**Published:** 2019-11-25

**Authors:** Xiaohuang Cao, Jianping Chen, Md. Nahidul Islam, Wanxiu Xu, Saiyi Zhong

**Affiliations:** 1 College of Food and Technology, Guangdong Ocean University, Zhanjiang 524000, China; caoxhfood@163.com (X.C.); cjp516555989@126.com (J.C.); 2 Department of Food Science, Aarhus University, Kirstinebjergvej 10, DK-5792 Aarslev, Denmark; nahidulislam.life@gmail.com; 3 College of engineering, Zhejiang Normal University, Jinhua 321004, China

**Keywords:** thermal processing, litchi fruit, phenolics, vitamin C, color

## Abstract

To examine the processing characteristics and high quality of an improved microwave vacuum drying system, litchi fruits were dried using intermittent microwave volumetric heating while microwave vacuum drying at 2 W/g was carried out for comparison; the intermittent microwave heating profiles were set as (1) 5 min drying-on, 5 min drying-off; (2) 5 min drying-on, 10 min drying-off; and (3) 5 min drying-on, 15 min drying-off. Energy consumption during drying was determined, and physicochemical properties such as moisture content, vitamin C, total phenolics, color, and sensory evaluation of dried products were assessed. In microwave vacuum drying, intermittent microwave volumetric heating was found to be energy-efficient (about 32 KJ/g to 45 KJ/g) and saved at least 31% of energy consumption compared with microwave vacuum drying as well as decreasing product browning. In addition, microwave volumetric heating had no substantial effects on sugar and protein contents, while antioxidants were affected significantly (*p* ≤ 0.05). Moreover, sensory evaluation showed that intermittent microwave-assisted vacuum drying (IMVD) increased the acceptance of the dried product compared with microwave vacuum drying (MVD).

## 1. Introduction

Litchi fruit (*Litchi chinensis* Sonn.) is a tropical fruit loved by consumers in China [1,2]. It has been cultivated in south China, and contains vitamin C, phenolics, and mixed sugars, which makes it healthy for body metabolism [3,4,5]. Litchi fruit has recently attracted the interest of processors and customers. If the concentrated fruit is transported as bulk, there is chance of massive losses due to spoilage and quality degradation [6,7,8,9]. Enhanced storage to facilitate transshipment could be achieved by dehydration, using methods such as sun drying, air drying, freeze drying, and microwave drying [7,8,9]. Sun-drying features the pollution of pests, microbes, and dust in an uncontrollable environment [8,9,10]. Whereas controlled dehydration such as freeze drying and air drying involve high costs and longer processing times, respectively [9,10]. On the other hand, microwave-assisted drying contributes to reducing time and cost; however, it may devaluate products in case of non-uniform heat, impairing product quality [5,11]. On the other hand, microwave vacuum drying allows balancing dehydration rate and product quality between vacuum-drying and microwave-drying [5,12]. Some researchers have evaluated the effect of microwave vacuum drying on the texture of pumpkin slices and found suitable to produce dried-and-crisp pumpkins [13,14]. However, microwave vacuum drying still induces browning of the fruits and vegetables because of its non-uniform heat [5,14]. To resolve this issue, different types of intermittent drying have been introduced, for example, intermittent microwave convective drying [15], intermittent heat pump drying [16], intermittent microwave convective drying [17], and intermittent microwave-convective air drying [18]. The strategy of applying intermittency allows time to transfer the moisture during the tempering period. Thus, quality degradation and heat damage can be minimized by applying intermittent drying [19,20]. In litchi fruit, the rapid transfer of moisture content is hindered, since it possesses 60–80% sugar solids, which bind water molecules [20,21,22,23]. This makes water diffusion difficult during drying; however, intermittent drying provides time intervals that promote diffusion.

The qualities of litchi include the amount of antioxidants, color, hardness, sugars, and other nutrients. Among these, color is easily degraded by the high temperature, which leads to browning [24,25,26,27]. Antioxidants such as vitamin C and phenolics are destroyed by the oxidizing reaction [27,28]. Therefore, a novel drying technique is necessary for obtaining high-quality dried litchi products. Intermittent microwave vacuum drying (IMVD) is considered an updated technique in high efficiency-quality processing. Interval microwave drying involves lower temperature, which implies less nutritional degradation [28,29,30,31]. However, little information on interval microwave vacuum drying is available, and no information can be found about IMVD of litchi fruits.

In our study, energy consumption, vitamin C, polyphenols, color, sugar, protein, water dynamics, and drying time during IMVD of litchi fruits were investigated [1,11,14]. The aims of this work were to present drying kinetics and energy consumption of IMVD and assess the quality of litchi fruits after IMVD.

## 2. Results and Discussion

### 2.1. Water Dynamics

**Figure 1:**
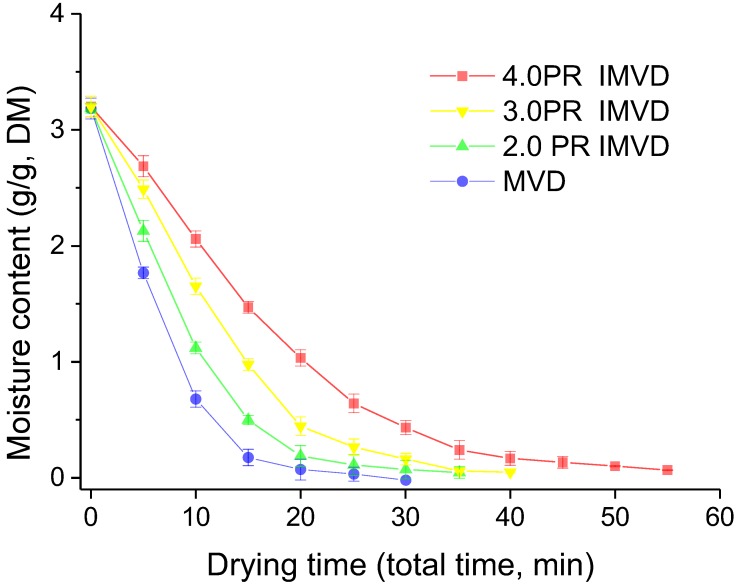
Water dynamics of samples dried by intermittent microwave vacuum drying and microwave vacuum drying; PR means pulse ratio and is calculated by dividing total time (drying-on plus drying-off) by drying-on time; MVD means microwave vacuum drying; IMVD means microwave vacuum drying with intermittent microwave heat.

Figure 1 shows the dehydration curves of dried samples at different times using IMVD and MVD. Different pulse ratios (2PR, 3PR, and 4PR) were calculated according to Equation (1). Dehydration curves are characterized by different dehydration kinetics related to cost and quality [5,22]. These drying kinetics are determined by temperature, properties of materials, and vacuum stage [11,23]. In the beginning, the drying rate was higher and gradually dropped for 10–20 min. The drying rate became constant after reaching the maximum drying rate; among the samples, that at 4.0 PR IMVD reached the constant level earlier. The reason for this phenomenon is that with the large accumulated volume of heat vapor of bulk water was accelerated faster [15,22]. IMVD reduced total drying-on time than MVD, although tending-zero drying in IMVD took longer than MVD. This means that additional intermittent drying could further decrease energy consumption. From Figure 1, it is obvious that intervals (off-drying) decreased the drying time while increasing the drying rate in IMVD of litchi fruits. The minimum value of drying time (4.0 PR) was 30 min since increased mass transfer of vapor in the tempering period.

### 2.2. Drying Time and Energy Consumption

Table 1 shows a comparison of the time and energy consumption of litchi fruits dried by IMVD and MVD. Energy consumption in IMVD was significantly lower than that in MVD. This means that increasing the interval (tempering time) enabled moisture transfer to the material surface by automatic processes. Results showed that IMVD saves at least 31% of energy consumption. Hence, a high drop in energy consumption in 4.0 PR was accompanied by long off-drying/tempering time, which allowed enough spontaneous diffusion. The process of automatic diffusion accounts for this phenomenon with vapor escaping from materials [24,25,26]. It was reported that high power (3 W/g) and low chamber pressure resulted in shorter drying time, suggesting that microwave vacuum drying may substitute freeze drying [11,25]. More studies confirmed that drying temperature and pulse ratio are key parameters of product quality and energy consumption [18,19]. IMVD is an attempt to achieve high quality and lower energy consumption in the drying of litchi fruits.

### 2.3. Color Evaluation

Table 2 presents color changes of litchi fruits dried by IMVD and MVD. Color values shifted to red and yellow after drying, which was significantly different (*p* ≤ 0.05) from fresh samples. This means that the thermal processing impaired the color of products and led to color charring. There was a significant difference in lightness between 4.0 and 2.0 PR dried samples; all IMVD dried samples were significantly different from the MVD sample in lightness. Interval heat decreased the occurrence of charring and browning. Several studies reported that browning formed in MVD decreased by relieves of thermal non-uniformity [5,11]. In case of yellowness, *b* increased with the decrease of PR, and a maximum *b* of 10.75 occurred in MVD dried samples. This phenomenon is ascribed to relieve of high-thermal, which hindered color degradation in the interval heating. Redness value *a* increased with the drop of PR in IMVD, and the maximum value of 5.56 occurred in MVD dried samples. The results showed a similar charring color during drying in accordance with *b* changes (Table 2).

Index ΔE value indicated that different PR dried samples were significantly different from each other in IMVD, and MVD dried samples were different from IMVD dried samples. This means different PR parameters affect browning differently; thus, IMVD provides different results regarding color. A high ΔE value indicates a large color change in the dried samples. A maximum ΔE of 12.11 was observed in MVD, in which the impaired color differed significantly from others (*p* ≤ 0.05). From Table 2, it can be seen that IMVD decreased the browning level with increasing pulse ratio to obtain more tempering for water vapor in low temperature.

### 2.4. Vitamin C

**Figure 2:**
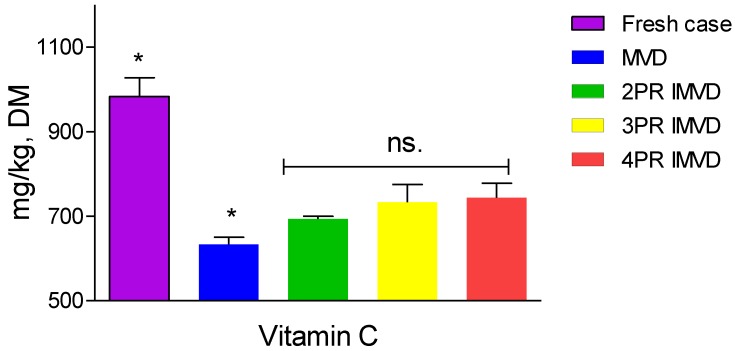
Vitamin C content of samples dried by intermittent microwave vacuum drying and microwave vacuum drying. Asterisk (*) means significant (*p* ≤ 0.05) difference; ns. means non-significant difference with *p* ≤ 0.05; PR means pulse ratio; MVD means microwave vacuum drying; IMVD means intermittent microwave vacuum drying.

Figure 2 presents the comparison of vitamin C content between fresh and dried litchi pulp. It shows a significant decrease of vitamin C content (*p* ≤ 0.05) after drying; meanwhile, significant differences emerged in vitamin C content between MVD and IMVD samples. This suggests that while MVD and IMVD both impaired the molecule of vitamin C, IMVD preserved a higher content of vitamin C than did MVD. From Figure 2, it can be seen that an average 60% of vitamin C was retained under IMVD, while MVD caused severe degradation. The exposure to high heat accounts for vitamin C degradation. Some researchers reported that the oxidase enzyme is responsible for vitamin C degradation; however, during drying, the loss of vitamin C is mainly due to non-enzymatic oxidation [22]. Duan et al. [1] reported 30–50% retention of vitamin C in MVD of litchi pulps, while IMVD conserved vitamin C at about 70% of fresh samples. When interval off-drying/tempering and on-drying are applied, the retention of vitamin C increased by about 10% compared to MVD. Thus, IMVD enables the promotion of vitamin C retention in dried litchi fruits in comparison with MVD.

### 2.5. Total Phenolic Compounds

**Figure 3:**
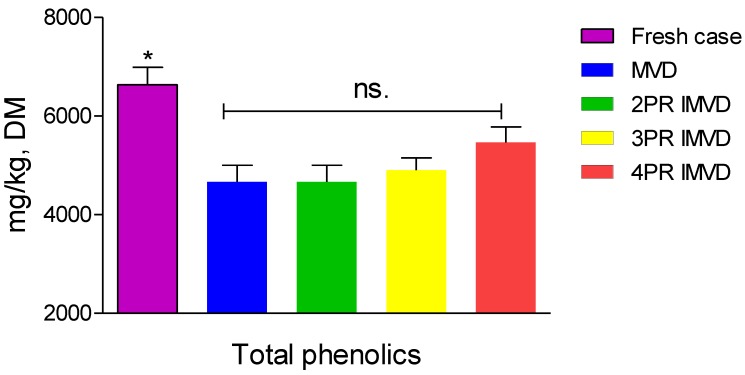
Total phenolic content of samples dried by intermittent microwave vacuum drying and microwave vacuum drying. Asterisk (*) means significant (*p* ≤ 0.05) difference; ns. means non-significant difference with *p* ≤ 0.05; PR means pulse ratio; MVD means microwave vacuum drying; IMVD means intermittent microwave vacuum drying.

Figure 3 presents the phenolic contents of samples dried by IMVD and MVD, and their comparison with fresh litchi fruits. Total phenolic compounds of dried samples decreased significantly (*p* ≤ 0.05) compared with the fresh sample. Results showed that interval volume heating in IMVD led to a higher amount of total phenolic compounds than MVD. In this sense, lower temperature and less time that implies less exposure to oxygen accounted for higher phenolic content. There was an average retention of 70–75% phenolic compounds in IMVD and MVD. There were no differences in phenolic contents of samples dried by the four drying methods. This might be caused to some extent by reducing sugar protected from oxidation [23,24,25].

### 2.6. Approximate Nutrition

Table 3 shows a comparison of protein, glucose, sucrose, fat, and acid content in IMVD and MVD dried litchi fruits. IMVD retained a higher content of glucose, fructose, and sucrose than did MVD. The decomposition could be due to higher oxidation in MVD. Higher temperature causes mass oxidation and decomposition to form charring substances [11,12].

From Table 3, the content of acid and protein decreased in MVD, and the content of fat decreased in IMVD. This could be due to more protein and sugar molecules undergoing non-enzymatic oxidation in MVD [32,33]. The low temperature in the off-dying time of IMVD retarded the occurrence of non-enzymatic browning reactions, which allowed a higher retention of sugar and protein in IMVD dried samples. The transformation of these sugars in litchi pulp improving taste originated from glucose and fructose degradation (Table 3). MVD led to significantly lower sugar (*p* ≤ 0.05) for browning and charring impaired nutrients.

### 2.7. Sensory Evaluation

Table 4 shows the sensory scores of different dried litchi fruit slices, including color, hardness, bitterness, and sweetness. These indicators are important for consumer preferences [5,11]. IMVD dried samples had better color and bitterness scores, while no differences in hardness and sweetness were found compared with MVD samples. MVD led to browning and increased bitterness, which lowered the evaluation scores. The main reason might be non-enzymatic oxidation resulting in darkness and bitterness [12,26]. This means that MVD leads to charring causing bitterness and forms sweet substances by high volume heat. Hardness in different drying profiles was not significantly different (*p* ≤ 0.05), and yielded a high score. Color scores for 4 PR dried slices were the highest in the sensory evaluation. This is in accordance with the results in Table 2. The reason might be that interval microwave heat deceased burning/charring. All sensory scores of dried samples were above 3.0, indicating that all dried products were acceptable.

The average scores increased with ascending PR values. Although the maximum PR (4.0) achieved high evaluation scores among dried samples, 3.0 PR dried samples obtained high average scores of 4.13. Thus, IMVD dried products achieved higher sensory scores for their high quality.

## 3. Materials and Methods

### 3.1. Materials

Fresh litchi fruit (10 kg) was carried from Guangzhou Origin Food Science & Technology Company Ltd., Guangzhou, China, to the College of Food and Technology, Guangdong Ocean University, using an ice-box within 24 h of harvesting. According to consumer preferences in China, litchi fruits (1 kg) were peeled and pitted along the fruit axis and then stored at 4 °C in a refrigerator.

### 3.2. Procedure

A sample of 10 g (4 mm thickness) was placed in a drying chamber (40 mm diameter, 80 cm height). Then, the vacuum pump was connected and the vacuum chamber was closed. Vacuum pressure was maintained at 0.05 MPa, and the microwave generator was operated at 2 W/g to evaluate the effects on selected parameters [1,11,14]. Drying intermittent profiles were set as (1) 5 min drying-on, 5 min drying-off; (2) 5 min drying-on, 10 min drying-off; and (3) 5 min drying-on, 15 min drying-off. In each drying scheme, the sampling interval lasted for 5 min, about 2 g of dried samples were taken out for determining moisture content, and then new samples (10 g sample) were loaded for drying until the next sampling time, i.e., the cycle of loading, drying, and sampling was repeated until the drying scheme was completed. The next sampling time was the previous drying time plus 5 min. In the final drying, retained dried samples were kept out for the quality test.

**Figure 4:**
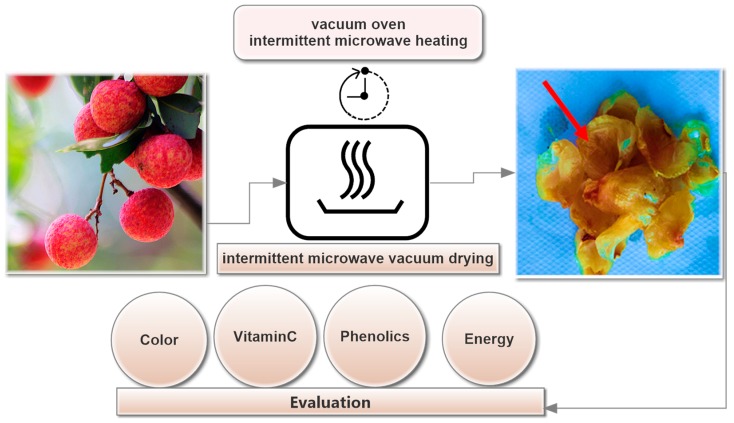
Flow of intermittent microwave heating of litchi fruits under vacuum drying.

The experimental flow diagram is shown in Figure 4. The initial moisture content was 78.2% (WB) and the end dried sample was obtained under 5% moisture and 0.35 water activity. The pulse ratio (PR) is expressed as follows:

(1) $$\mathrm{PR}=\frac{t_{on}+t_{off}}{t_{on}}$$

where t_on_ is the “on” time and t_off_ is the “off” time of drying (min). PR is an important parameter related to energy consumption as well as drying kinetics.

### 3.3. Moisture Content

Dried samples (1 g) were randomly placed into the constant weighing bottle. Samples were dried using an air-drying oven at 105 °C until constant mass or until changes occurred at the third decimal place. The water content of litchi fruit was calculated using Equation (2) [26]:

(2) $$\mathrm{MC}=\frac{M_{t}-M_{1}}{M_{t}}\times 100\%$$

where MC represents the wet base moisture content (g/g); $M_{1}$ represents the dry matter mass (g); $M_{t}$ is the fresh mass (g) of litchi fruit.

### 3.4. Determination of Vitamin C

Grounded samples of 2 g were transferred to a flask (500 mL) with 50 mL distilled and deoxygenated water. 10 mL of 20% sulphuric acid solution and 2 mL of 0.5% starch solution were added to the sample. The added mixture was titrated against 0.01 M I_2_ solution until blue color emerged without fading for 30 seconds. The titrated mass was calculated for the vitamin C content (mg of vitamin C/kg, DM) [1,21].

### 3.5. Total Phenolic Compounds

Samples of 2 g of litchi pulps were smashed mechanically and scattered in 20 mL of 80% acetone. Then, 10 mL of 5 M hydrochloric acid (HCl) was added for hydrolysis in a magnetic stirrer at 80 °C for 4 h. The hydrolyzed solution was filtered using an additional 50 mL of acetone. The extracted phenolic compounds (1 mL) were tangled with 5 mL distilled water and 5 mL of Folin & Ciocalteu’s reagent [22]. After that, 15 mL 7% Na_2_CO_3_ solution was added and incubated for 10 min. Then, the incubated solution (1 mL) from 26 mL mixture was analyzed using a spectrophotometer at 760 nm. The standard substance was gallic acid. Concentration of total phenolics was represented as mg/kg of gallic acid equivalents in dry matter (DM).

### 3.6. Color Measurement

The surface color of samples was measured randomly using a NR20XE precision handheld colorimeter with an aperture of 20 mm (3nh, Shenzhen, China). Three parameters were recorded in the five average measurements under natural light. *L*, *a*, and *b* of fresh pieces was considered as ideal values; *L* is lightness, *b* is yellow, and *a* is redness [23,24,25]. Before measurement, the instrument was calibrated with a whiteboard. From the measured values, ΔE was calculated using Equation (3).

(3) $$\Delta E=\sqrt{{\left(L_{1}-L_{0}\right)}^{2}+{\left(a_{1}-a_{0}\right)}^{2}+{\left(b_{1}-b_{0}\right)}^{2}}$$

where *L*_0_, *a*_0_, *b*_0_, and *L*_1_, *a*_1_, *b*_1_ represent the values of the fresh samples and dried samples, respectively.

### 3.7. Approximate Nutrition

The nutrients in litchi pulps consist mainly of sugars, protein, fat, and minerals. Protein was determined by the Kjeldahl method [7,24]. Total acids were determined by acid-base titration, and total fat was measured using the Soxhlet extraction method [27,34]. Total sugars include glucose, fructose, and sucrose, which were determined as previously described [1].

Dried fruit slices weighing 0.5 g were grounded and dissolved in 45 mL water in a 50 mL volumetric flask. The dissolved solution was then filtered with 0.2 µm filter paper and prepared for chromatographic analysis. The conditions for liquid chromatography were as follows; cation exchange resin columns (7.8 mm, 300 mm), resin particles: HW-65F resin (30–60 µm); mobile phase: distilled water; flow rate: 0.5 mL/min, and temperature: 80 °C. 10 mg/mL of filtered mixture was injected into system sampler. By comparison of peak time and peak area with standard glucose, fructose, and sucrose, the content of these three sugars was determined.

### 3.8. Energy Consumption

Energy consumption for each dryer parts was measured with an electric energy analyzer (DTS634, CHNT, Leqing, China) by inserting a wire line of a microwave generator. Total energy consumed was divided by sample mass without consideration of ohmic resistance.

### 3.9. Sensory Evaluation

The sensory panel consisted of 20 experienced panelists (10 men and 10 women, between 20 and 30 years old). Randomly chosen, 12 dried samples (2 g) were sent for assessing. Scores on color, hardness, bitterness, and sweetness were collected for analysis. A 5-point scale was used for scoring: 5 = excellent, 4 = good, 3 = acceptable, 2 = fair, and 1 = unacceptable. The evaluation was performed at a room temperature of 25 °C with two fluorescent lamps (100 W).

### 3.10. Data Analysis

Experimental data were analysed using SPSS software (SPSS 20.0, IBM, Chicago, IL, USA). Mean significance differences were determined by Duncan’s multiple range test. A confidence level of 95% (*p* ≤ 0.05) was applied; all diagrams are drawn with Origin 8.0 software. We calculated mean values and standard error of mean; all experiments and measurements were done in triplicate unless otherwise stated.

## 4. Conclusions

We investigated the energy consumption of IMVD of litchi fruit and resulting product quality. Intermittent micro-volumetric heat was first induced into vacuum drying, and its merit was qualified to further progress in food processing. Drying kinetics, antioxidant substances, processing time, energy consumption, and sensory evaluation were investigated regarding IMVD of litchi fruits. The IMVD mode of 3.0 pulse ratio achieved optimal quality and consumed less energy; thus, this mode is suggested for application.

In this work, reduced browning, lower cost, and shorter drying time were recorded in IMVD as well as increased retention of nutrient content and antioxidant substances. IMVD involves interval volume heating of drying material instead of continuous volume heating, and thus produces better quality dried product at lower cost. Thermal charring dropped markedly in IMVD using pulse volumetric heat. MVD still has the drawback of causing burning of the material, while IMVD resolved this issue. Therefore, IMVD would be an effective alternative to further develop the drying process of litchi fruit.

## Figures and Tables

**Table 1:** Drying time and energy consumption of samples dried by intermittent microwave vacuum drying and microwave vacuum drying.

Drying	Total Time (min)	On-Drying Time Accumulation (min)	Off-Drying Time Accumulation (min)	Energy Consumption (KJ/g)
4.0 PR IMVD	55	15	40	33.5 ± 1.15 ^b^
3.0 PR IMVD	40	15	25	32.3 ± 1.11 ^c^
2.0 PR IMVD	35	20	15	45.5 ± 1.25 ^b^
MVD	30	30	0	65.7 ± 1.21 ^a^

Different letters indicate significant differences (*p* ≤ 0.05) in energy consumption; PR means pulse ratio; MVD means microwave vacuum drying; IMVD means intermittent microwave vacuum drying.

**Table 2:** Color indexes of samples dried by intermittent microwave vacuum drying and microwave vacuum drying.

Drying/70 °C	PR	*L*	*a*	*b*	ΔE
Fresh		52.72 ± 1.44 ^a^	−4.45 ± 0.11 ^a^	5.55 ± 0.32 ^a^	
On 5 min/off 15 min	4	29.44 ± 1.35 ^b^	3.31 ± 0.15 ^b^	8.25 ± 0.44 ^b^	6.11 ± 0.31 ^a^
On 5 min/off 10 min	3	27.72 ± 1.13 ^b,c^	3.12 ± 0.21 ^b^	9.55 ± 0.51 ^c^	7.53 ± 0.42 ^b^
On 5 min/off 5 min	2	25.71 ± 1.24 ^c^	4.44 ± 0.22 ^c^	10.75 ± 0.55 ^d^	9.25 ± 0.45 ^c^
MVD		22.62 ± 1.18 ^d^	5.56 ± 0.25 ^d^	12.81 ± 0.58 ^e^	12.11 ± 2.43 ^d^

Different letter indicates significant differences (*p* ≤ 0.05) in a column; PR means pulse ratio; MVD means microwave vacuum drying.

**Table 3:** Approximate nutrition of litchi pulps processed by different drying schemes.

IMVD	Approximate Nutrition of Dried Litchi Fruits Using IMVD (g/100g)					
PR	Protein	Glucose	Fructose	Sucrose	Fat	Acid
2.0	2.40 ± 0.05 ^a^	27.65 ± 0.55 ^a^	21.25 ± 0.23 ^b^	15.77 ± 0.52 ^a^	2.57 ± 0.03 ^a^	2.22 ± 0.05 ^a^
3.0	2.55 ± 0.07 ^a^	28.77 ± 0.21 ^a^	21.27 ± 0.55 ^b^	12.52 ± 0.71 ^b^	2.60 ± 0.03 ^a^	2.24 ± 0.01 ^a^
4.0	2.52 ± 0.03 ^a^	28.62 ± 0.53 ^a^	23.17 ± 0.32 ^c^	13.72 ± 0.73 ^b^	2.70 ± 0.05 ^a^	2.21 ± 0.03 ^a^
MVD	1.57 ± 0.03 ^b^	25.11 ± 0.45 ^b^	18.68 ± 0.55 ^a^	11.55 ± 0.41 ^c^	2.75 ± 0.02 ^a^	3.12 ± 0.01 ^b^

Different letter indicates significant differences (*p* ≤ 0.05) in a column; PR means pulse ratio; MVD means microwave vacuum drying; IMVD means intermittent microwave vacuum drying; acid value represents citric acid.

**Table 4:** Sensory evaluation of dried litchi fruit slices.

Parameters	IMVD			
	MVD	2 PR	3 PR	4 PR
Color	3.62 ± 0.12 ^a^	4.12 ± 0.15 ^a^	4.44 ± 0.12 ^b^	4.54 ± 0.13 ^b^
Hardness	4.21 ± 0.12 ^b^	4.24 ± 0.12 ^a^	4.32 ± 0.12 ^a^	4.41 ± 0.11 ^a^
Bitterness	2.12 ± 0.15 ^a^	3.45 ± 0.15 ^b^	3.25 ± 0.12 ^b^	3.57 ± 0.13 ^b^
Sweet	4.21 ± 0.13 ^a^	4.15 ± 0.17 ^a^	4.25 ± 0.10 ^a^	4.67 ± 0.12 ^a^
Acid	4.11 ± 0.20 ^a^	4.34 ± 0.15 ^a^	4.42 ± 0.17 ^a^	4.51 ± 0.16 ^a^
Average score	3.64 ± 0.12 ^a^	4.06 ± 0.13 ^a^	4.13 ± 0.15 ^b^	4.34 ± 0.13 ^b^

Different letters in the same row indicate significant differences (*p* ≤ 0.05). Scores were as follows: 5 = excellent, 4 = good, 3 = acceptable, 2 = fair, and 1 = unacceptable. PR means pulse ratio; MVD means microwave vacuum drying; IMVD means vacuum drying with intermittent microwave heat.
